# Esophageal adenocarcinoma in Barrett’s esophagus after sleeve gastrectomy: Case report and literature review

**DOI:** 10.1016/j.ijscr.2018.10.015

**Published:** 2018-10-12

**Authors:** Lionel El Khoury, Rosa Benvenga, Rodolfo Romero, Regis Cohen, Joel Roussel, Jean-Marc Catheline

**Affiliations:** Department of Digestive Surgery, Centre Hospitalier de Saint-Denis, 2 Rue du Docteur Delafontaine, 93205, Saint-Denis, France

**Keywords:** Laparoscopic sleeve gastrectomy, Gastroesophageal reflux disease, Barrett’s esophagus, Esophageal adenocarcinoma

## Abstract

**Introduction:**

: Sleeve gastrectomy (SG) has become one of the most dramatically increasing bariatric procedures worldwide due to its excellent results and impact on the obesity pandemic. Morbid obesity is known to increase the risk of esophageal adenocarcinoma. However, the evolution of gastroesophageal reflux disease (GERD) along with Barrett’s esophagus (BE) after SG is of concern since there is little data available. We present a case of esophageal adenocarcinoma after SG with known intestinal metaplasia on preoperative gastroscopy.

**Case presentation:**

: We report a case of a 55 years old female patient who presented lower esophageal adenocarcinoma three years after complicated SG with known preoperative BE without dysplasia detected by gastroscopy. Multidisciplinary decision suggested treatment by endoscopic mucosectomy. The endoscopic control at one year did not highlight tumoral recurrence but still BE without dysplasia.

**Discussion:**

: Literature review regarding the evolution of BE after SG is poor and the relation between SG and the development of subsequent esophageal cancer isn’t clear yet.

**Conclusion:**

: Preoperative endoscopy should be performed in order to detect GERD, BE, and potential carcinomas of the upper gastrointestinal tract before undergoing bariatric surgery. The long-term monitoring after SG is essential.

## Highlights

• Literature evidences regarding the evolution of Barrett’s esophagus after sleeve gastrectomy is poor and the relation between sleeve gastrectomy and the development of subsequent esophageal cancer isn’t clear yet.
• Preoperative upper endoscopy should be performed in order to detect gastroesophageal reflux disease, Barrett’s esophagus, before undergoing bariatric surgery. Post operative monitoring of the upper gastrointestinal tract after sleeve gastrectomy is essential.

## 1 Introduction

Compared to general population, people with morbid obesity have an increased risk of gastro-esophageal reflux disease (GERD), esophagitis, and subsequent related complication as Barrett’s Esophagus (BE) and esophageal cancer [1]. Bariatric surgery is the only treatment of pathological obesity and effective prevention of obesity related comorbidity. However, the effect of bariatric surgery on preexisting GERD, or newly developed GERD, mainly after sleeve gastrectomy (SG), is still controversial. SG is the most performed procedure worldwide. On the one hand, SG induces an alteration of gastroesophageal junction along, increases intragastric pressure, and divides gastric sling fibers, hence inducing GERD. On the other hand, weight loss after surgery is supposed to improve of reflux symptoms [2]. Despite the fact that GERD is not an absolute contraindication to LSG, many surgeons prefer Roux-en-Y gastric bypass (RYGBP) whenever severe uncontrollable reflux and/or BE are present. RGYBP mechanisms of diverting bile away from the esophagus, decreasing acid production in the gastric pouch and reducing volume of acid reflux, are well known [3]. Little is known about the evolution of BE after SG and the incidence of esophageal adenocarcinoma. Gagner [4] noted that after over 20 years of performing duodenal switch procedures (including sleeve), there is no report yet of esophageal carcinoma in more than 100,000 patients operated all over the world [4]. We presented a case of lower esophageal adenocarcinoma after SG in a patient with a preoperative BE.

## 2 Case report

**Fig. 1:**
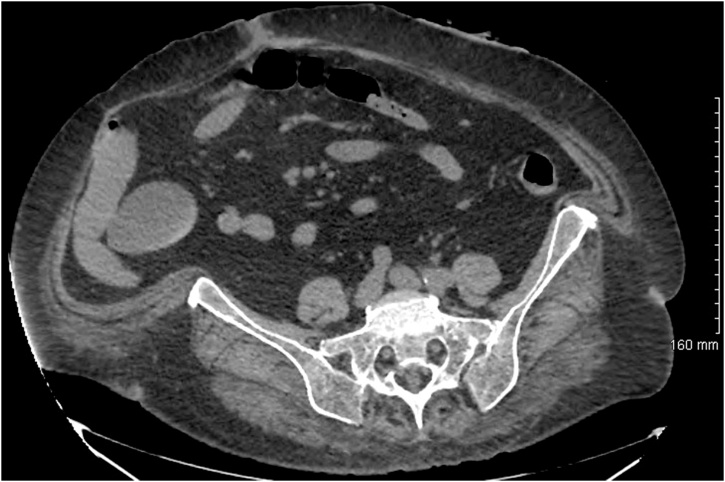
Preoperative abdominal CT with enormous recurrence of incisional hernia along the right iliac fossa.

A 55 years old female obese patient with Body Mass Index (BMI) of 42 kg/m [2] (120 kg, 1.70 m) with past medical history of hypertension, type 2 diabetes mellitus, dyslipidemia, arthritis. She had also a past surgical history of open appendectomy, incisional hernia treated with intraperitoneal mesh, complicated with right colic erosion and fistula requiring right colectomy. She arrived from another hospital with multiple median and transverse abdominal scar incisions and recurrence of an enormous incisional hernia in her right iliac fossa (Fig. 1). No tobacco or alcohol intoxications were noted. Preoperative upper fibroscopy revealed BE along 2 cm in height without dysplasia on biopsy. A multidisciplinary team decided to perform SG initially followed by incisional hernia repair. Open SG was performed. No intraoperative complication occurred. Two weeks later a gastric leak appeared requiring surgical reoperation and drainage by Kehr tube. Fistula was dried up six months later.

**Fig. 2:**
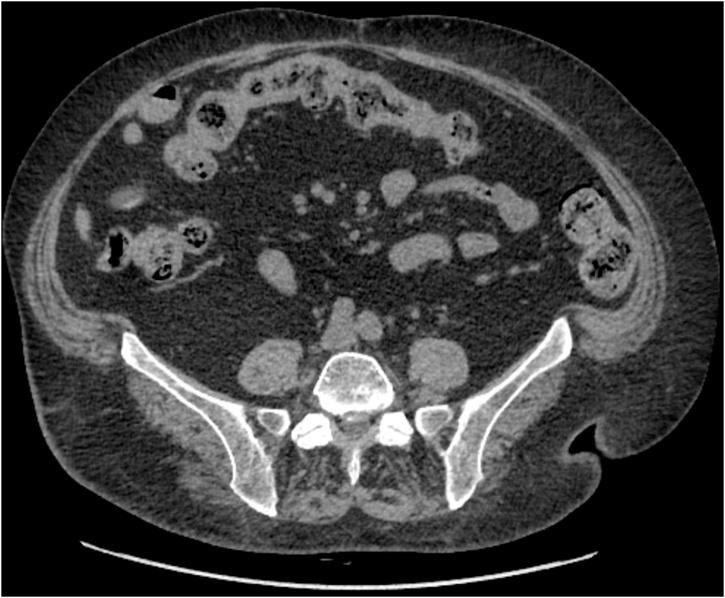
Abdominal CT scan after definitive cure of the incisional hernias.

Seventeen months after SG and a 40 kgs weight loss, the median and right iliac hernias were repaired with pre-aponeurotic polypropylene mesh along with abdominal dermolipectomy. The latter operation was complicated with cutaneous dehiscence, which required treatment by vacuum therapy. One year later, a supraombilical incisional hernia was repaired successfully using a preaponeurotic subcutaneous polypropylene mesh (Fig. 2).

**Fig. 3:**
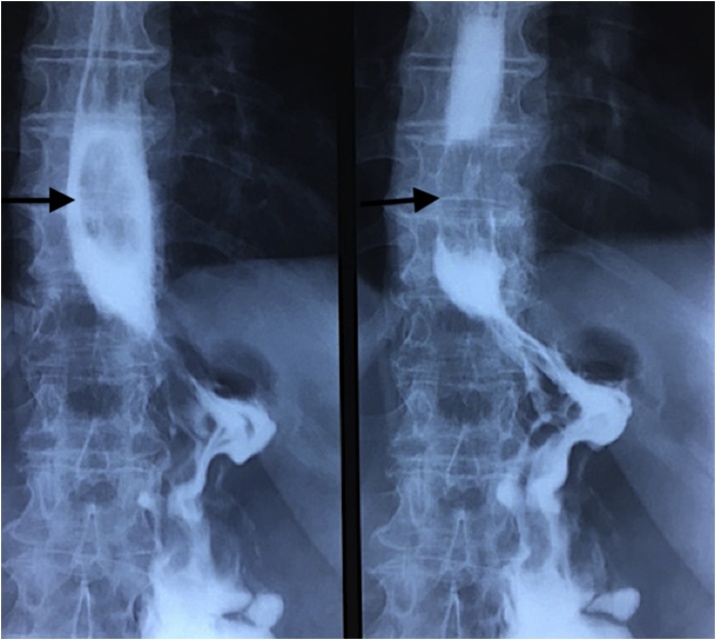
Upper gastrointestinal opacification showing lower esophagus lacunar image three years after sleeve gastrectomy.

**Fig. 4:**
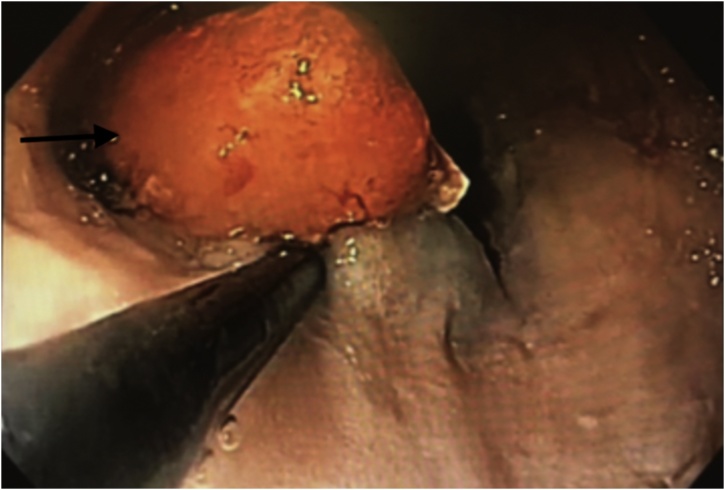
Upper endoscopic mucosectomy of the polypoid lesion of the lower esophagus.

Three years following SG, the patient presented with complaining of persistent vomiting over several months. Upper digestive tract opacification showed lacunar image on the lower esophagus (Fig. 3). Endoscopy revealed 5 cm pseudo-polyp neoformation located 30–35 cm from the dental arches. Upper endoscopic ultrasound described a supra-cardial pediculate hypoechoic lesion of 2 cm in diameter without peritumoral lymph nodes. After multidisciplinary discussion, endoscopic mucosectomy was done (Fig. 4). Biopsy showed well-differentiated intra-epithelial adenocarcinoma arisen in BE with safe surgical margins and without lymphatic embolus or perineural sheathing; classified as pT1 with negative HER2 expression. Work up being negative for hepatic or extrahepatic disease, close supervision and follow up of the patient with serial gastroscopy were suggested later on.

## 3 Discussion

We report the first case of esophageal adenocarcinoma three years after SG in a patient with BE. Different epidemiological studies supported the existence of a relationship between obesity and increased prevalence of cancer, and particularly esophageal adenocarcinoma [5]. Other factors have been suggested, such as genetic factors, BE, Helicobacter Pylori infection, tobacco smoking, alcohol consumption, dietary factors, male hormones, and medications [5]. Bariatric surgery, by its action on obesity, should be related to a decrease in tumor incidence [6]. However, it cannot completely prevent cancer occurrence given that the pathophysiology of cancer has not been fully understood yet.

A systematic review [7] of 28 articles reported 33 cases of gastroesophageal cancer after bariatric surgery including RYGB, gastric banding, and vertical banded gastroplasty. In this review SG was not mentioned. To the best of our knowledge, only a few cases of esophageal cancer after SG have been reported. One case occurred four months after SG in a patient who did not undergo preoperative upper endoscopy [8]. Another case reported by Sohn [9] described esophageal adenocarcinoma 2.5 years after SG also without previous endoscopic evaluation. In a recent publication, Wright [10] described esophageal adenocarcinoma five years after SG in a patient with normal previous preoperative gastroscopy. Our case is the only one with BE without dysplasia detected before SG. The main pathophysiology of esophageal adenocarcinoma following SG could result from chronic GERD, which could induce intestinal metaplastic changes.

There is no available data in the literature describing the evolution of preexisting BE after SG. But many reports describe the positive effect of RYGB on regression and even cure of BE [3]. Thus many authors [3,4] recommend RYGB for obese patients with reflux-related complications, including severe esophagitis and BE. Our patient was multioperated with a complex surgical history and RYGB was deemed too difficult. It has not been possible to establish whether SG had an impact on the natural history of BE and its evolution to cancer since the patient was asymptomatic. The effect of SG on GERD is still controversial. Braghetto [11] noted an increase in GERD after SG varying from 2.1% and 27.5% of the 130 and 167 patients respectively with no preoperative GERD. Tai [2] mentioned 34.9% increase of prevalence of GERD in 67 patients operated by the same surgeon after a one year follow up. The supposed mechanisms responsible for this increase of GERD after SG would be an alteration of the angle of His due to surgery itself; hypotony of the lower esophageal sphincter after division of muscular sling fibers; decrease of gastric volume and consequent increased intragastric pressure; worsening of hiatal hernia; probable decrease of ghrelin, hence dysmotility and neofundus formation [12]. On the other hand, Melissas [13] reported a 5% decrease in GERD after SG. Weiner [14] also noted a 20% decrease in GERD after surgery in 120 patients followed along five years. The mechanisms supporting these arguments are accelerated gastric emptying, decreased abdominal obesity, decreased acid production, and restoration of His angle over time [12]. This contradiction adds complexity to the literature research for concluding whether SG is or not a suitable option for patients with preoperative GERD. Reflux is usually related to hiatal hernia, thus the relationship between SG and hiatal hernia should also be stressed on. Many studies reported reparation of hiatal hernia in conjunction with SG. This showed encouraging results, as mentioned by Sucandi [15] whose study reported 50% approximately cured from reflux. Moreover, Lyon [16] found an improvement in 262 patients after combining hiatal repair with SG, and concluded that reflux and hiatal hernia should no longer be seen as a contraindication for SG as many authors would suggest. Daes [17] demonstrated that hiatoplasty with SG assume good results since 94% of the 382 patients studied became asymptomatic after surgery. However, Sieber [18] noted worsening of GERD after sleeve gastrectomy in spite of concomitant cure of hiatal hernia whenever necessary, thereby adding further confusion in interpretation of literature results. Those latter results could be due to undetected pre- and intraoperative small hiatal hernias, which could grow bigger over time and lead to reflux. However, concomitant repair of hiatal hernia with SG could be a good option for reducing GERD after surgery.

If SG complicates natural evolution of BE, its preoperative detection appears mandatory in order to prevent esophageal cancer. The incidence of BE after SG was first described by Braghetto [19] who found 1.2% of *de novo* intestinal metaplasia at five years following surgery. A recent publication [20] noted 15% of BE at ten years starting from 0% preoperatively. Analysis of these data call for systematic preoperative endoscopy [20]. Accordingly to European Guidelines, we perform a routinely preoperative gastroscopy and not after surgery. There are no data about endoscopic surveillance in asymptomatic patients after bariatric surgery.

## 4 Conclusion

An increased risk of esophageal cancer after SG could not be evidenced today. However physicians should be aware of the increased prevalence of GERD given that the young age of sleeve patients could represent a risk. We suggest RYGB for patients with BE or GERD and even converting SG to RYGB whenever GERD is severe and/or BE is of concern. The role of preoperative and postoperative endoscopy could be crucial, especially in asymptomatic patients. More average risk based studies and recommendations for postoperative endoscopic surveillance are needed. Long-term monitoring after SG is essential.

## Conflicts of interest

No conflict of interest.

## Funding

No funding.

## Ethical approval

The patient have given her informed consent for this publication.

It is exemption from ethical approval because it is an observation report after the current care.

## Consent

She gave us her consent.

Written informed consent was obtained from the patient for publication of this case report and accompanying images. A copy of the written consent is available for review by the Editor-in-Chief of this journal on request”.

## Author contribution

Each author have participated sufficiently in the work to take public responsibility for appropriate portions of the content. All authors met all of the following criteria:

- Substantial contributions to the conception or design of the work; or the acquisition, analysis, or interpretation of data for the work; AND

- Drafting the work or revising it critically for important intellectual content; AND

- Final approval of the version to be published; AND

- Agreement to be accountable for all aspects of the work in ensuring that questions related to the accuracy or integrity of any part of the work are appropriately investigated and resolved.

JMC and RR operated the patient. LEK and RB wrote the first draft of the manuscript. JMC, RC, and JR wrote the final draft of the manuscript. JR made the corrections in English. All authors have read and approved the final report.

## Registration of research studies

Not applicable.

## Guarantor

On the behalf of all author I am the guarantor.

Régis Cohen.

## Provenance and peer review

Not commissioned, externally peer reviewed.
